# Modeling Body Mass Variation: Incorporating Social Influence into Calculations of Caloric Intake and Energy Expenditure

**DOI:** 10.1371/journal.pone.0111709

**Published:** 2014-11-04

**Authors:** Ana María Hernández-Hernández, Rodrigo Huerta-Quintanilla

**Affiliations:** Departamento de Física Aplicada, Centro de Investigación y de Estudios Avanzados del Instituto Politécnico Nacional, Mérida, Yucatán, México; Monash University, Australia

## Abstract

Variations in individual body mass and composition have long been a key focus in the health sciences, particularly now that overweight and obesity are considered as public health problems. We study a mathematical model that describes body mass variations which are determined by the energy balance between caloric intake and total energy expenditure. To calculate the change in caloric intake and energy expenditure over time, we proposed a relationship for each of these quantities, and we used measured values that are reported in the literature for the initial conditions. To account for small variations in the daily energy balance of an individual, we include social interactions as the multiplication of two terms: social proximity and social influence. We observe that social interactions have a considerable effect when the body mass of an individual is quite constant and social interactions take random values. However, when an individual's mass value changes (either increases or decreases), social interactions do not have a notable effect. In our simulation, we tested two different models that describe the body mass composition, and it resulted that one fits better the data.

## Introduction

Overweight and obesity have become worldwide health problems because they cause several diseases [1]–[4]. In response, various disciplines, particularly the health sciences, have focused on variations in human body mass and composition [5]–[9], particularly on factors that lead to increases in human body mass [10]–[15]. Mathematical models and their numerical solutions can be used to quantify changes in body mass and are therefore useful tools for studying body mass. Caloric intake and total energy expenditure are two of the most important and complex factors that should be quantified [16], [17]. Our aim in the present study is to propose a set of formulas as a function of individual body mass and implement them to estimate how body mass varies over time. Then, a random variation is introduced to represent variations in the caloric intake and energy expenditure from those for the daily routine of an individual; this variation provides a simple method of accounting for social interactions.

## Results/Discussion

**Figure 1:**
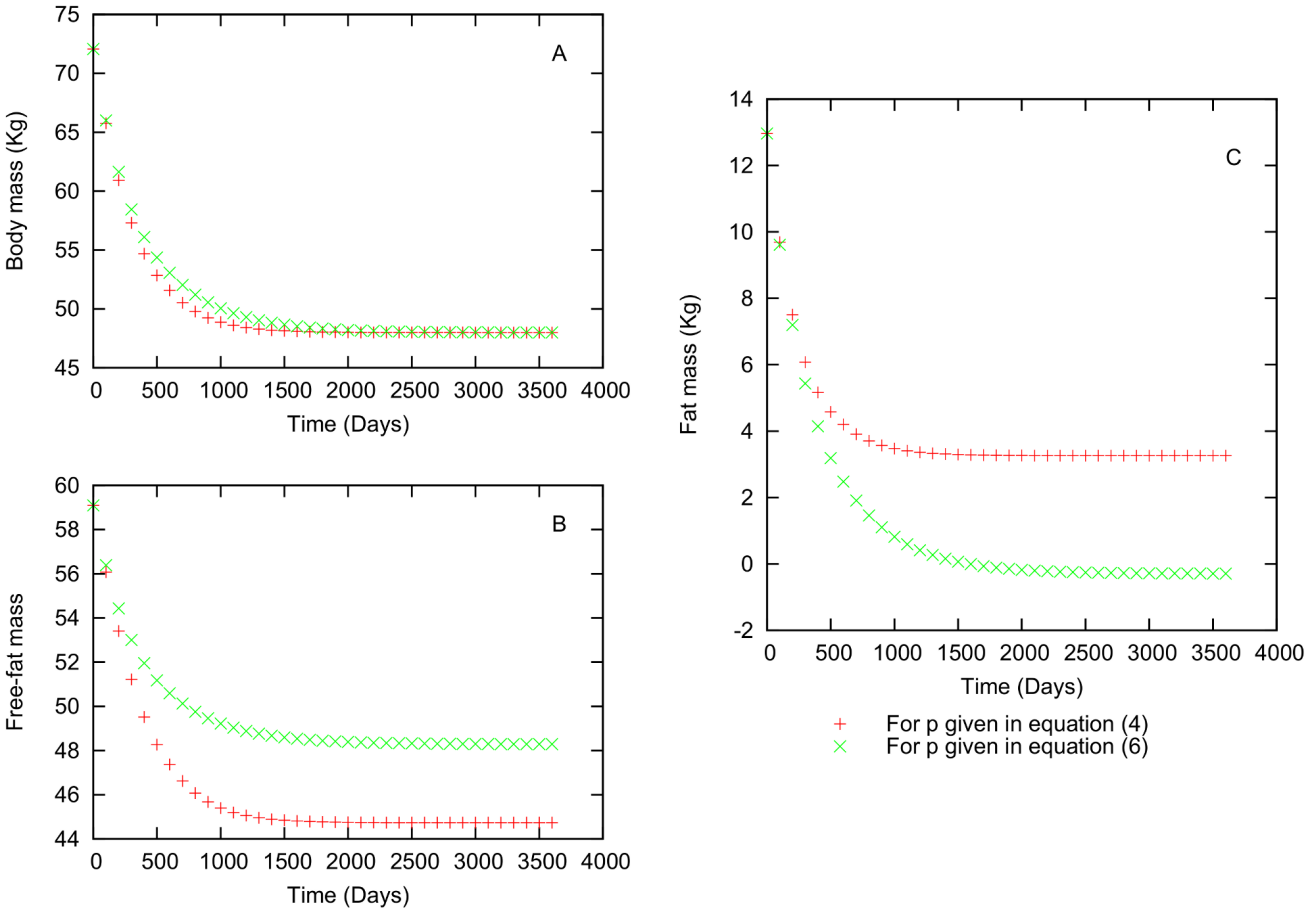
Difference between the Forbes and Hall relationships for an individual who is losing weight. Characteristics: 30.43 years old, 72.06 kg initial body mass, 12.96 kg initial body fat mass, 59.09 kg initial lean mass, and height = 1.88 m.

**Figure 2:**
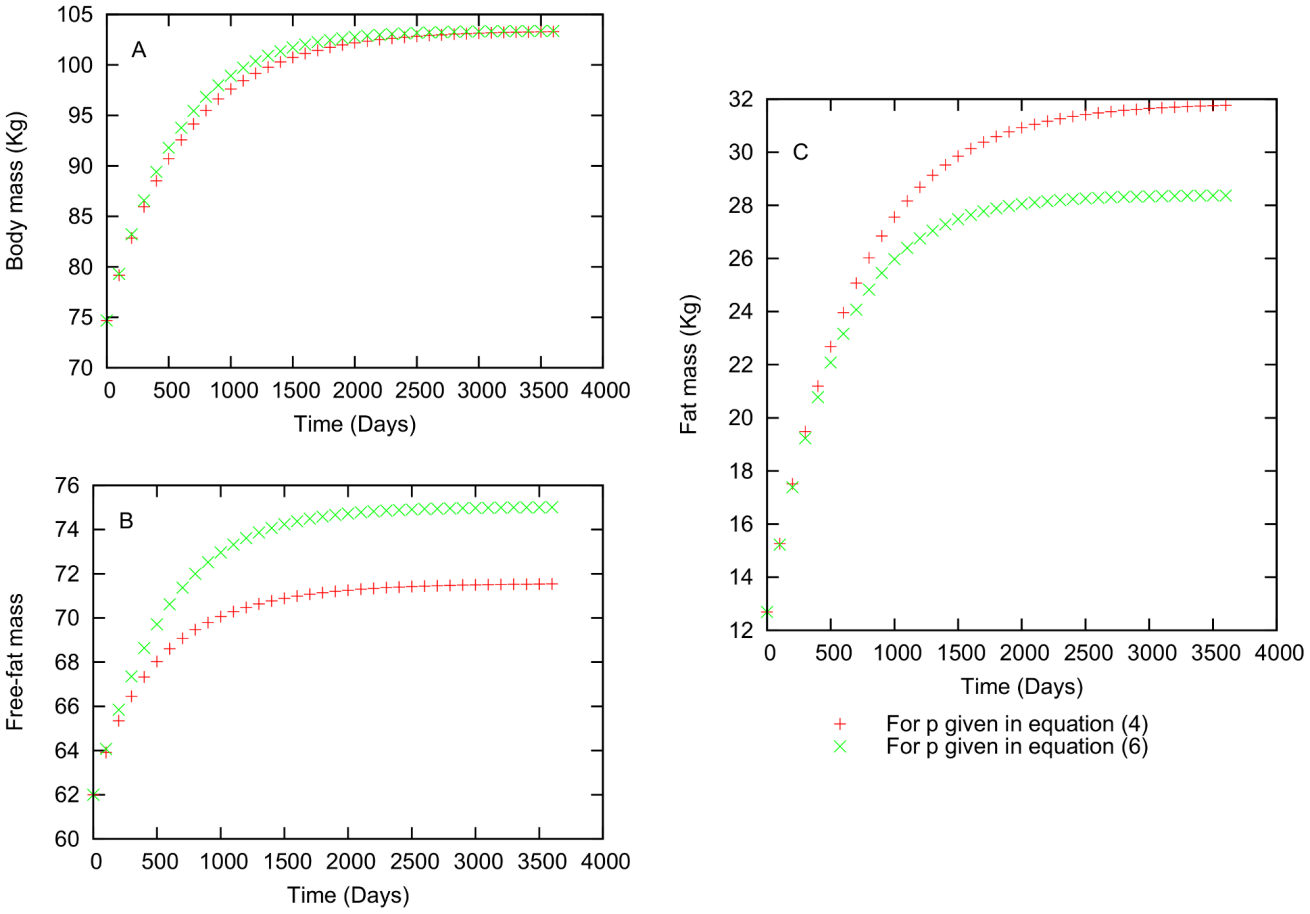
Difference between the Forbes and Hall relationships for an individual who is gaining weight. Characteristics: 20.26 years old, 74.69 kg initial body mass, 12.69 kg initial body fat mass, 61.99 kg initial lean mass, height = 1.89 m.

We introduce values for the initial caloric intake and total energy expenditure to the equation system of Hall and Chow [18]. We choose initial values for intake and total energy expenditure from energy distributions based on an extensive Food and Agriculture Organization (FAO) study [19]. These energy distributions have a dependency on individuals weight, age and sex. Initial values for mass, height and body fat percentage, which are chosen by sex, are also required for each individual. [20], [21]. Once all the initial values are given, the simulation can begin. We performed several simulations to compare the results of the numerical solutions for equations (2) and (3) using the two expressions for p given by equations (4) and (6) [22], [23]. The first simulations were conducted to test the expresions for p in equations (4) and (6) and choose one of these equations for our next simulations. We found that one of the equations provides a more satisfactory description of the body mass variation. In this case we determined that the Forbes relationship produces better results. In figures 1 and 2, we show two examples of individuals with different initial conditions. To test these formulas we performed simulations with large changes in body weight. In figure 1, we show the change in the total body (A), lean (B) and fat (C) masses with respect to time for a simulated male individual with the following characteristics at the initial time 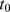: age(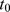) = 30.43 years, mass 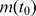 = 72.06 kg, fat mass 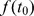 = 12.96 kg, lean mass 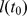 = 59.09 kg, intake 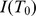 = 2821.48 Kcal, total energy expenditure 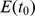 = 3248.75 kcal, height h = 1.88 meters and 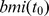 = 20.37 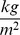. The value for variable 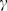 for this individual is chosen randomly, and is 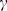 = 0.32 in the present case. As shown in figure 1A, there is no significant difference in the simulations using equation (4) and the simulations using equation (6). However, in figures 1B and 1C, we noticed a large difference between the two cases. In fact, in figure 1C, using equation (6) predicts negative values for the fat mass. These negative values are due to the way p changes. In the equation (4) p reaches an steady value only when F becomes constant. In the other hand, in the equation (6) the contribution to p comes from both values, F and L, and it is the latter which is responsible of the negative values.

In figure 2, we show a simulation of a male individual with the following characteristics: age(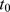) = 20.263 years, m(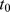) = 74.69 kg, f(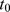) = 12.69 kg, l(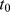) = 61.99 kg, 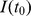 = 3288.08 kcal, 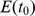 = 29991.30 kcal, h = 1.89 meters and bmi(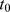) = 20.75 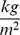. The value for 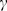 is chosen randomly, and for this case 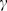 = 0.44. Figure 2A shows that, there is not a substantial difference in total body mass values that are found using equations (4) and (6). However, in figures 2B and 2C, we can observe that the values of fat and lean mass values obtained using the two relationships for p differ appreciably. In equation (6), lean mass increases more quickly than fat. However, for equation (4) we have the opossite case. From figure (1), we conclude that Forbes equation (4) is more adequate for our next simulations.

### Body mass changes for different values of 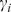


In the simulations, we consider 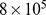 individuals to obtain the most representatives variations for random initial conditions. We chose two representative examples: a man who loses weight and a woman who gains weight. Other examples, such as a woman who loses weight and a man who gains weight, are also possible. We chose these examples for their representative curves that show how body mass, caloric intake and total energy expenditure vary over time.

**Figure 3:**
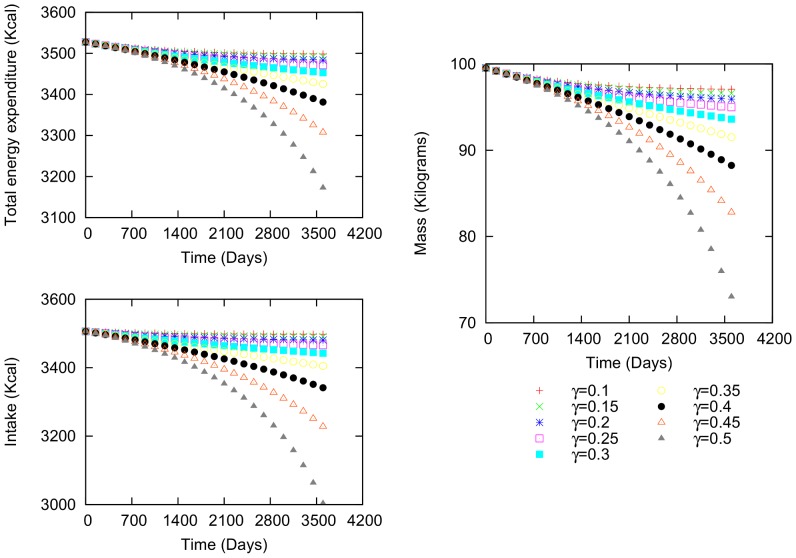
Body mass, intake and total energy expenditure variation for a male. Characteristics: 38.44 years of age, 99.52 kg initial body mass, 3506.32 kcal initial intake and 3527.51 kcal total energy expenditure.

The first example is a man who is 38.44 years old and 1.76 m in height (figure 3). His initial values are 99.52 kg weight, 37.11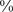 body fat percentage, 3506.32 kcal intake and 3527.51 kcal total energy expenditure. The variations in mass, intake and energy expenditure over time have the same functional shape. As 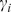 becomes greater than zero, the intake variation increases over time, and consequently, the body mass variation also increases. Thus, as the value of 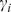 increases, more time is required to reach a stable value. In addition, the difference between the caloric intake and energy expenditure is negative, causing the individual to lose weight.

**Figure 4:**
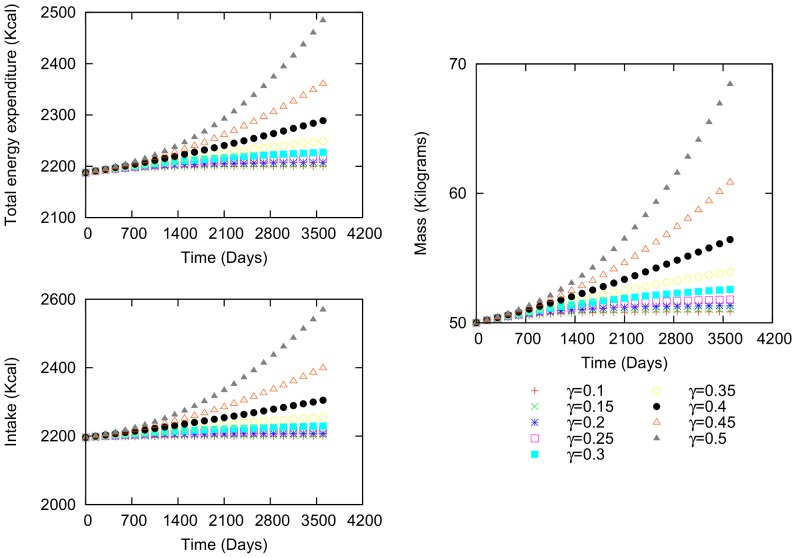
Body mass, intake and total expenditure variation for a female. Characteristics: 34.54 years of age, 50.01 kilograms initial body mass, 2196.22 kcal initial intake and 2187.62 kcal total energy expenditure.

The second example is a woman, who is 34.45 years old and 1.54 m in height (figure 4). Her initial values are 50.01 kg weight, 20.40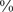 body fat percentage, 2196.22 kcal intake and 2187.62 kcal total energy expenditure. Because her energetic difference is positive, she gains weight. Again, the functional shape of the variations in mass, caloric intake and total energy expenditure is similar. As 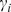 increases in value, more time is required for the mass, caloric intake and total energy expenditure values to reach the maximum values, that is, when the intake and expenditure have the same value and the energetic difference is zero.

Although we use basic mathematical equations, our results are comparable to those of previous studies [9], [12], [18]. However, we do not believe that the trend in body mass variation would be that consistent when small time steps are used. Thus we introduce the hypothesis that the social environment affects intake and total energy expenditure.

### Modeling social interactions as energy noise

The simulations use one day as a single time increment, and we introduce a small amount of noise to simulate the daily situations that are beyond an individual's control, such as social interactions. These interactions vary depending on daily circumstances and can influence an individual's caloric intake and total energy expenditure. Therefore we use equations (13) and (14), which include a term that we introduced to describe social interactions. To begin studying the effect of this social term [24], we use three relevant cases. The first case (i) uses a fixed value for social proximity 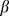 with a variation in the social influence 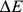. In the second case (ii), a fixed value is used for the social influence 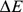 with 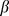 varying between 0 and 1.

Human interactions are irregular, and the type of situations that people encounter vary, which can also cause randomness in these interactions. To account to these factors we propose a third case (iii) in which random values are assigned to both the social proximity and the social influence. In the following simulations, we take 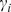 = 0.3, which is the average value of the uniform distribution, to simplify the calculations.

**Figure 5:**
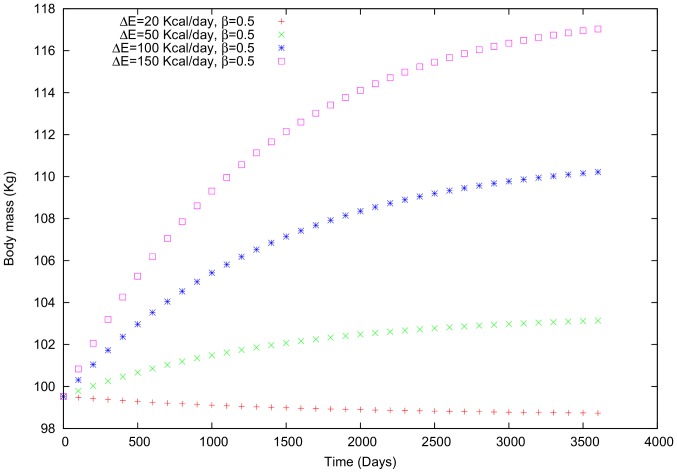
Body mass in a male individual for whom 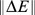 is variable and 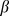 = 0.5.

**Case i**: To estimate the effects of social energetic differences on an individual, the social proximity is fixed at 0.5, and 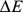 is varied between 20 and 150 kcal per day. In the first example, which involves a man who loses body mass due to personal intake, including the social term has a positive effect on his body mass (figure 5). The social influence (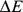) is associated with the caloric intake and total energy expenditure but does not have a role in the individual's daily routine; instead, the social influence is a type of noise that is linked to the social interactions.

**Figure 6:**
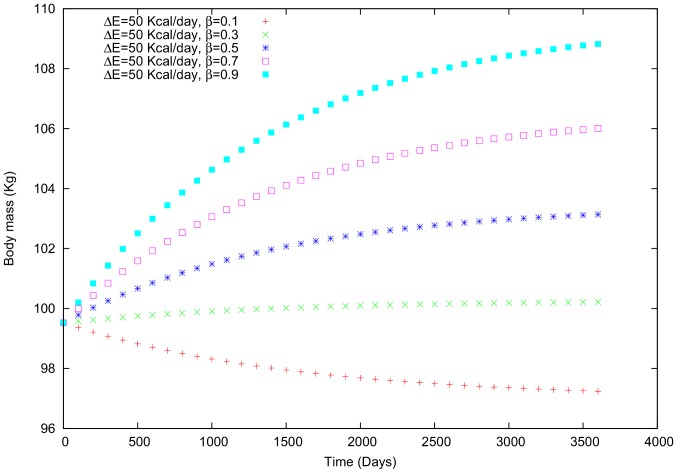
Body mass in a male individual for whom 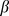 is variable and 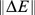 = 50 Kcal/day.

**Case ii**: In this case, a fixed value for social influence (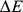) is used and 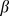 is varied from 0 to 1. For example, in figure 3, a positive value is used for the social influence (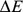 = +50 kcal/day; figure 6). The social proximity is correlated with the interaction nearness; that is, larger values of 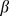 (i.e., values closer to 1) correspond to a stronger relationship between this individual and the other individuals with whom s/he interacts. This nearness can be physical or social, but we do not differentiate between the types of nearness in these cases.

Comparing cases (i) and (ii) using the first example (figure 3) reveals that varying the social influence (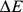) causes a more pronounced increase in body mass than just varying 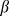. In other words, although the individual is motivated to lose weight in this case, as indicated by his negative personal energetic difference (figure 3), interacting with individuals whose routine leads to an increase in body mass (we use a positive sign in equations (15) and (16)) undermines his attempt to lose weight. Indeed, as 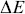 increases, the body mass variation becomes positive and the mass increases. In case (ii), increased interactions with other individuals (i.e., 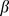 approaching 1) again makes the variation in body mass positive.

**Figure 7:**
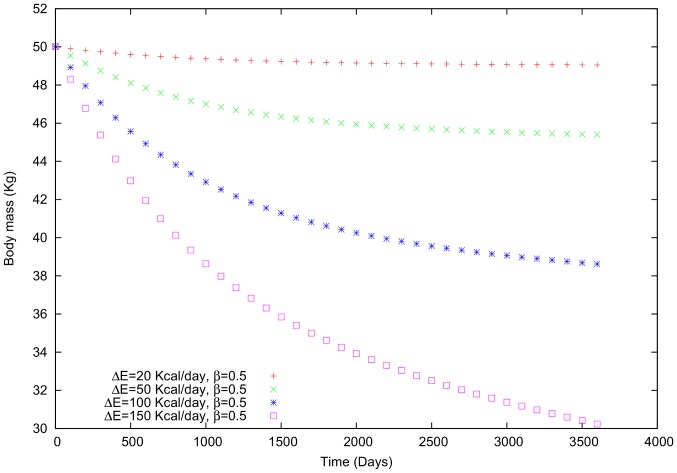
Body mass in a female individual for whom 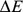 is variable and 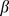 = 0.5.

**Figure 8:**
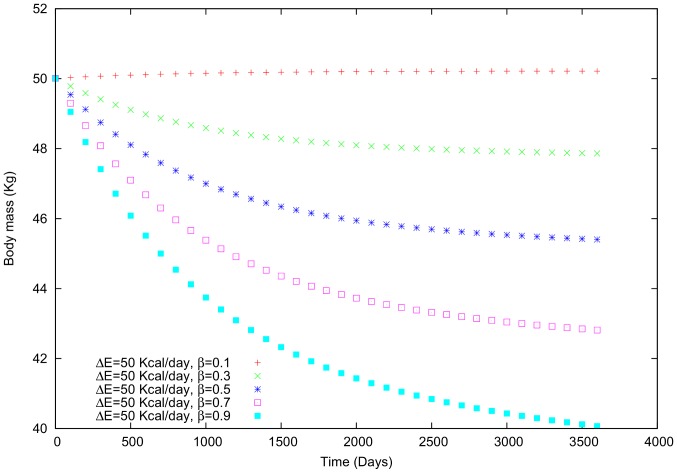
Body mass in a female individual for whom 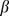 is variable and 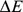 = 50 Kcal/day.

Comparing (i) and (ii) using the second example (figure 4), reveals that varying 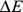 (in this case, we use a negative sign) generates a greater variation in body mass than does increasing 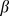 (figures 7 and 8). Although the individual's tendency is to gain weight slightly, the social interaction leads to a negative variation in body mass. In other words, if a person who wants to gain weight interacts with others who want to lose weight, it will be much more difficult for the former individual to attain her goal. In general, we noticed that gaining (or losing) and maintaining weight will become even more challenging in the case of strong social interactions. [25]

**Figure 9:**
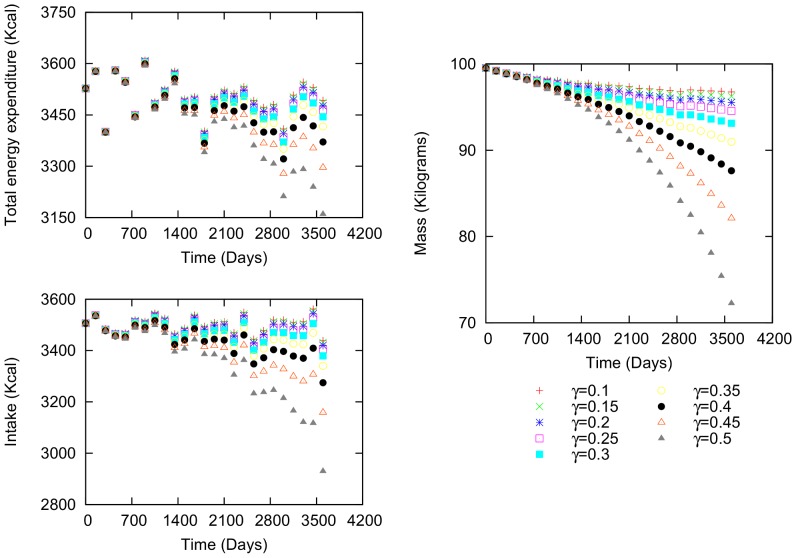
Variation in body mass, intake and total energy expenditure for a male with social noise. Characteristics: 38.44 years of age, 99.52 kg initial body mass, 3506.32 kcal initial intake and 3527.51 kcal total energy expenditure.

**Figure 10:**
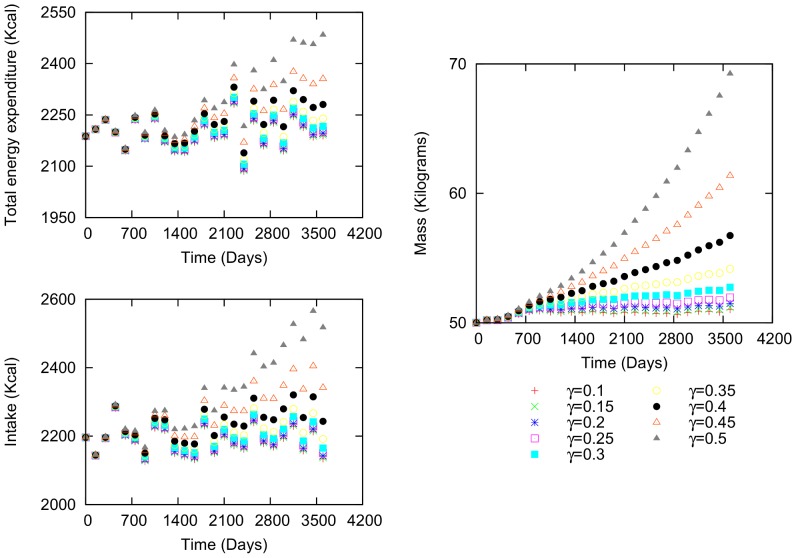
Variation in body mass, intake and total expenditure for a female with social noise. Characteristics: 34.54 years of age, 50.01 kg initial body mass, 2196.22 kcal initial intake and 2187.62 kcal total energy expenditure.

**Case iii**: We use a random value for the social energy influence 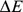 that is selected from a uniform distribution of points in the range of [0,300] kcal/day, a random sign for the social interaction in equations (15) and (16), and we vary 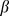 uniformly from (0,1), in the first example (figure 3). These parameters produce small variations over time that are more irregular than those in figure 3 (figure 9). The body mass varies, but the variations are more pronounced in intake and total energy expenditure. When the same conditions are used in the second example (figure 4), the same variation patterns occur (figure 10).

**Figure 11:**
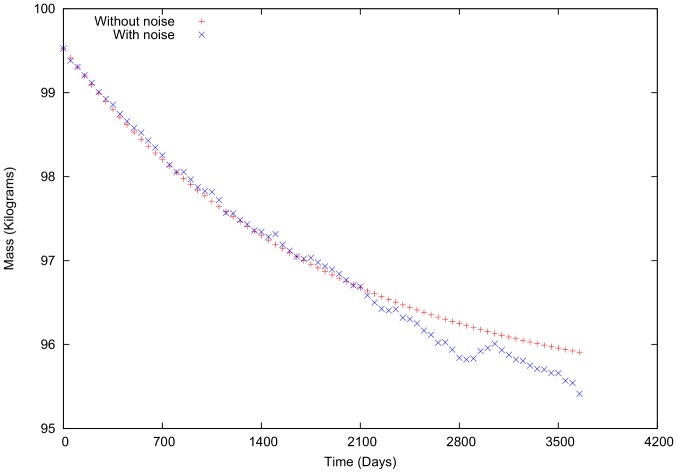
Body mass variation with and without social terms in intake and total energy expenditure for a male, 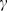 = 0.3.

During the first time steps in both cases (with and without social interaction), the body mass change is more pronounced and follows similar patterns in both. However, as the body mass approaches a stable mass value, the fluctuations are much larger for the case in which social interaction is included (figure 11). This difference occurs because as body mass approaches a stable value, the personal energetic difference tends toward zero. As a result, the social interactions cause large variations and thus lead to caloric intake and expenditure values that differ from those for the individual's daily routine.

**Figure 12:**
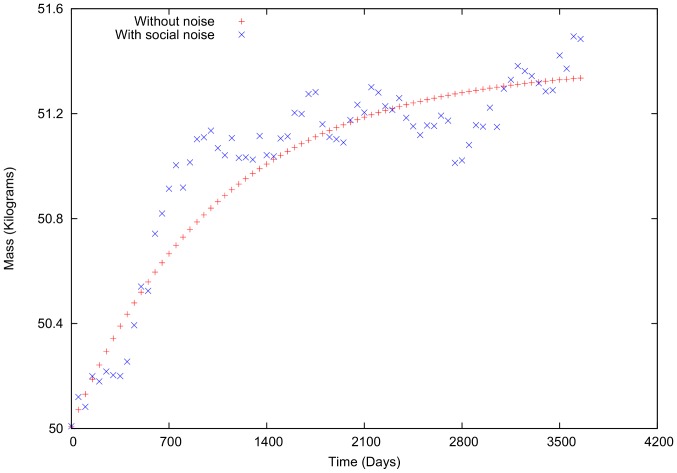
Body mass variation with and without social terms in intake and total energy expenditure for a female, 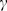 = 0.3.

Using the second example (figure 4) and the same conditions as above, the body mass variation is positive (figure 12). Both cases (with and without social interaction) have similar tendencies; however, in comparison to the previous example, the variations are less pronounced. In other words, the personal energetic difference (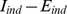) approaches zero more rapidly. Consequently, the social interactions have larger effects across the entire time interval. It becomes clear that if the social interaction mean value is not zero, there would a tendency for the body mass to change accordingly. We expect that with the introduction of networks in these calculations, this tendency will be the result of the individual's interaction with its neigborhood, depending on the average mean value of the neighbor's energetic differences.

Simulations similar to those above provide a theoretical foundation that provide suggestions about how the human environment can affect body mass. The regular variation patterns (figures 3 and 4) exhibit no peaks, but the social interactions are important and play a vital role in the variation. Several studies have addressed the effects of social interactions by considering kinship, friendship, gender, age and other factors [25]–[27]. However, no definitive conclusions have yet been reached as to how social interactions directly affect important variables such as an individual's intake and total energy expenditure. Introducing social interaction into the present equation system using energetic terms helps to elucidate how a network of individuals whose body masses increase and decrease affect an entire population. This tool could be useful in studying how social interactions can modify the percentages of individuals in a network who have obesity, overweight or low weight, as well as the average BMI of the network. Future work can be performed to explain the effect of social networks on variations in mass.

## Methods

Many analyses of the variations in individual body mass have been developed to predict changes. Recently, mathematical models have been developed that can make predictions that cover a large number of variables that affect human body mass. The model proposed by Chow and Hall [18] mainly focuses on the differences between caloric intake and total energy expenditure; this model is capable of incorporating new terms such as the term we introduce to describe social interactions. Initially, this model is based on the law of energy conservation:
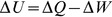
(1)where 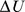 is the change in the energy reserved or accumulated in the body; 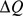 is the variation in caloric intake; and 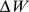 is the variation in total energy used. Using the mathematical model of Chow and Hall leads to the following equations:
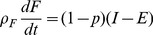
(2)




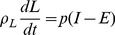
(3)where 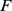 is the individual's fat mass; 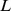 is lean mass (including all non-fat tissues: organs, bones, muscle, etc.); 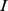 is the caloric intake; 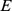 is the total energy expenditure; 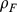 is the energy density associated with body fat; 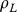 is the energy density associated with lean mass; and 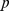 is calculated as follows:
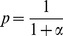
(4)where 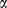 is calculated by
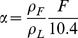
(5)The caloric intake (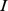) and total energy expenditure (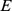) are not constant values; instead, these values change over time. Equation (5) was formulated by Forbes [12] for adult women, although we use this equation as an approximation in the model for both sexes. To adjust the model, we solve equations (2) and (3) numerically and propose relationships for the caloric intake and total energy expenditure (for the latter, we use equations given by the FAO [19]). In our study, we are dealing with total mass and define the total mass of an individual as 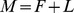. However, there is another mathematical form for 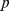 that is given in equation (4) and proposed by Hall in [13].
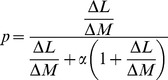
(6)where 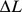 is the difference between the lean mean at time t and time t-1, 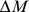 is the difference between the body mass at time t and t-1, and 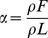. In this work, we compare the Forbes and Hall expressions for 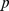 using computer simulations.

### Energy intake and total energy expenditure

Various computational models have been proposed to calculate intake [8], [10], [14], [15] and thus to analyze body mass variations in large numbers of individuals. We introduce a new theoretical model designed to identify a way to include dynamic change in caloric intake. We must properly describe the large variations in body mass among individuals. Considering this, we introduce a new parameter 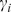 (which depends on the individual) that would be related to how fast the body mass of an individual reaches equilibrium after significant variations in the caloric intake values. The caloric intake values are then related to the parameter 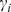 through the following equation that is proposed for either gaining or lossing weight:
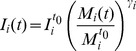
(7)where 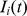 is the caloric intake of an individual 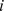 at time 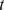; 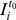 is the caloric intake of the individual 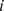 at the initial time 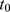; 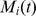 is the body mass of an individual 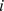 at time 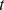; and 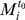 is the body mass of an individual 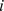 at time 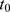. To determine the range of 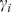, we performed 8 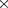 10^5^ simulations and analyzed the data. Comparisons with the data showed in various papers in the literature [9], [18], [20] indicated that the optimum 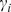 values are consistent with the literature values for regular variations of 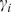 in the interval (0.1, 0.5). In our simulation, we change the value of 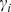 within the range from 0.1 to 0.5 by increments of 0.05. The parameter 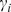 was introduced with the only intention to model the individual's intake dynamically since very often in the literature the intake values are taken as constants. Additionally, with the introduction of this parameter, we can assign to each individual a different gamma value. Doing so, we can model each of the individual's metabolic rate, for instance, taking into account for particular differences among them.

The total energy expenditure is calculated by a well-known expression: (used in [21] for body mass changes)
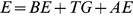
(8)where 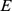 is the total energy expenditure; 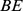 is the average basal expenditure [19]; 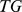 is thermogenesis; and 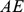 is the energy expenditure for activity. Using the initial conditions just mentioned, the total energy expenditure at time 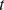 is calculated as (for an individual 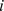)
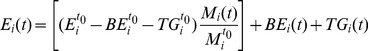
(9)where 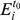 is the total energy expenditure at the initial time 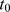; 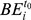 is the basal expenditure at the initial time 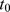; thermogenesis is taken as 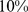 of the intake 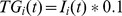 [22]; 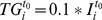 is the thermogenesis at the initial time 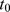; and 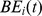 is the basal expenditure at time 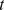. The expenditure for activity (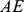) is shown in terms of the initial conditions so that only known variables are used:
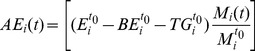
(10)


### Introducing the social interaction to energy terms

Body mass variations can sometimes be induced by other people, depending of the type of social influences exerted on an individual. We want to describe social interactions by adding a random term to the intake and energy expenditure equations. Our intent is to model the small daily variations in an individual that are caused by stimuli that lead him/her to change intake or energy expenditure from his/her normal habits. To this end, we define intake as
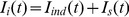
(11)where 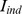 is the individual caloric intake, and 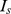 is the socially-induced caloric intake. The total energy expenditure takes the form
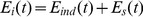
(12)where 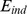 is the total individual energy expenditure, and 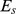 is the total socially-induced energy expenditure. These social terms, 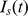 and 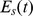, are modeled in the following manner
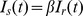





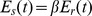
where the variable 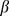 is taken as the strength of the social influence [24], which we term social proximity. 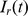 and 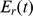 are the socially induced intake and expenditure, respectively. As a result, equations (2) and (3) take the following forms
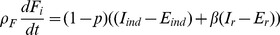
(13)

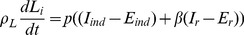
(14)where the energetic difference induced by others is termed the *social influence* (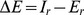). The product of the social proximity and the social influence will give us the total *social interaction* [24]. Using the energy distributions obtained from documented data in FAO studies, we found that the energy difference of an individual was within the range of [-300∶300] kilocalories. This change correspond to a daily variation of 10 to 20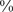 of the caloric intake, depending on the individual. Therefore, equations (13) and (14) can be approximated as
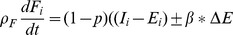
(15)




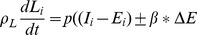
(16)where the social proximity parameter 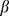 takes random values from a uniform distribution of points in the (0.0, 1.0) range, the social influence 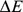 take random values from a uniform distribution of points in the (0, 300) range and the sign is chosen randomly.

## Conclusions

The present simulations indicate that, in general, social interactions have a greater effect on body mass variation when individual body mass is mantained near a stable value, see figure 12. This finding is expected because the social interaction is introduced in a random manner. However, as shown in figure 11, the social interactions are relevant but even are more significant when the individual energetic differences (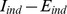) becomes close to zero. Comparing our results with other calculations [8], [10], [18], we can infer that the parameter 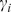 introduced in equation (7) is useful for this type of simulations, because it allows for the inclusion of dynamic variation in caloric intake; this parameter is sufficient for our purpose and allows the equation system to reproduce variations in the body mass of an individual for different periods of time. In addition, the parameter can be used to incorporate other individual-dependent effects, such as phenotype. The mass variation of an individual, who is part of a social network, can also be explored for different types of networks. This would be part of our future work.
